# High-Throughput Sequencing of MicroRNA Transcriptome and Expression Assay in the Sturgeon, *Acipenser schrenckii*

**DOI:** 10.1371/journal.pone.0115251

**Published:** 2014-12-15

**Authors:** Lihong Yuan, Xiujuan Zhang, Linmiao Li, Haiying Jiang, Jinping Chen

**Affiliations:** Guangdong Public Laboratory of Wild Animal Conservation and Utilization, Guangdong Entomological Institute/South China Institute of Endangered Animals, Guangzhou, China; University of Nordland, Norway

## Abstract

Sturgeons are considered as living fossils and have very high evolutionary, economical and conservation values. The multiploidy of sturgeon that has been caused by chromosome duplication may lead to the emergence of new microRNAs (miRNAs) involved in the ploidy and physiological processes. In the present study, we performed the first sturgeon miRNAs analysis by RNA-seq high-throughput sequencing combined with expression assay of microarray and real-time PCR, and aimed to discover the sturgeon-specific miRNAs, confirm the expressed pattern of miRNAs and illustrate the potential role of miRNAs-targets on sturgeon biological processes. A total of 103 miRNAs were identified, including 58 miRNAs with strongly detected signals (signal >500 and *P*≤0.01), which were detected by microarray. Real-time PCR assay supported the expression pattern obtained by microarray. Moreover, co-expression of 21 miRNAs in all five tissues and tissue-specific expression of 16 miRNAs implied the crucial and particular function of them in sturgeon physiological processes. Target gene prediction, especially the enriched functional gene groups (369 GO terms) and pathways (37 KEGG) regulated by 58 miRNAs (*P*<0.05), illustrated the interaction of miRNAs and putative mRNAs, and also the potential mechanism involved in these biological processes. Our new findings of sturgeon miRNAs expand the public database of transcriptome information for this species, contribute to our understanding of sturgeon biology, and also provide invaluable data that may be applied in sturgeon breeding.

## Introduction

Sturgeons (order: Acipenseriformes, infraclass: Chondrostei), are believed to have separated from other teleosts over 250 MYA, and are referred as living fossils providing a key phylogenetic position for evolutionary studies on vertebrates [1]. As different degrees of ploidy are resulted from the multiple and independent duplication events [2], [3], sturgeon species have been divided into two groups (approximately 120 and 240 chromosomes), and the division of the species into two groups of either diploid/tetraploid or tetra/octoploid is still unclear [4], [5]. A previous study showed that microRNAs (miRNAs) in *Arabidopsis thaliana* evolved with a process of genome-wide duplication followed by dispersal and diversification, and the duplicated copies of miRNAs may acquire new functionality during their evolution [6]. A later study proved that almost all of miRNAs of Rainbow trout (*Oncorhynchus mykiss*) have been retained as duplicated copies [7]. These results indicated that, by chromosome duplication, new miRNAs in sturgeon may be evolved, and thus reflect new functions. Hence, in-depth study of sturgeon miRNAs would improve the knowledge of sturgeon ploidy, and also evolutionary history of sturgeons and vertebrates.

MiRNAs are small RNA molecules (18–25 nt), and have been identified to play key roles in directing transcriptional and post-transcriptional activity of mRNAs, and are thus involved in the regulation of multiple biological processes such as differentiation and development, immune response, reproductive system development and gametogenesis [8], [9]. The study of the sturgeon miRNAs and their interactions with target mRNAs will provide further insight into these physiological processes of sturgeon. RNA-seq, an ultrahigh-throughput sequencing technique has greatly increased our understanding on the complexity of eukaryotic mRNA and small RNA transcriptomes [10], including those of non-model species, which have neither genomic background nor miRNA data in miRbase [11], [12].

In the present study, we carried out the first high-throughput sequencing analysis on miRNAs of five key tissues (liver, spleen, muscle, heart and brain) of the Amur sturgeon *Acipenser schrenckii,* an endangered and important economic sturgeon species, using the RNA-seq technique on the Illumina TruSeq sequencing platform. Combining with the expression level validation of miRNAs by microarray and stem-loop real-time PCR, our study aims to discover sturgeon-specific miRNAs, investigate the expression pattern and illustrate the potential role of miRNAs and their targets on sturgeon biological processes.

## Materials and Methods

### Ethics statement

The protocol was approved by the Committee on the Ethics of Animal Experiments of the Guangdong Entomological Institute, which also incorporates the South China Institute of Endangered Animals. Sturgeon individuals were immerged in the water with 10^−4^ (v/v) Eugenol about 1–3 minutes for euthanasia, following the AVMA guidelines (2013) for use [13]. All efforts were made to minimize suffering.

### Sample and RNA preparation

The five tissues (liver, spleen, muscle, heart, brain) of a 5-month-old Amur sturgeon, *Acipenser schrenckii*, from the Engineering and Technology Center of Sturgeon Breeding and Cultivation of Chinese Academy of Fishery Science (Beijing, China) were collected. Total RNA were extracted from five tissue samples separately with RNAiso reagent (TaKaRa, Japan) according to the manufacturer’s instructions. RNA concentration was measured using Qubit RNA Assay Kit in Qubit 2.0 Flurometer (Life Technologies), and RNA purity was assessed using the Nano Photometer spectrophotometer (IMPLEN). Total RNA of five tissues (3 ug each) were pooled, and RNA integrity was inspected using the RNA Nano 6000 Assay Kit of the Bioanalyzer 2100 system (Agilent Technologies). The pooled RNA sample with RNA Integrity Number (RIN) = 8.3 met the needs of TruSeq transcriptome/small RNA library construction and sequencing, which hereafter referred to as ASY transcriptome/small RNA library.

### Transcriptome references sequencing

As no genomic sequences specific to *A. schrenckii* are available on public database, we firstly carried out the de novo transcriptome sequencing and assembling by Illumina TruSeq platform. 3 ug pooled RNA was used as mRNA library construction using Illumina TruSeq RNA Sample Preparation Kit (Illumina) following manufacturer’s recommendations. Briefly, mRNA was purified from 3 ug pooled RNA by using poly-T oligo-attached magnetic beads. After the first and second strand cDNA synthesizing, DNA fragments were converted into blunt ends, adenylated the 3′ ends, and then ligated with Illumina PE adapter oligonucleotides for hybridization. Then, cDNA fragments with length >200 bp were purified with AMPure XP system (Beckman), and those ligated with adapters on both ends were selectively enriched using Illumina PCR Primer Cocktail in a 10 cycles PCR reaction, and the products were purified again by AMPure XP system and quantified by Agilent 2100 bioanalyzer. Subsequently, the cluster of index-coded samples was generated using TruSeq PE Cluster Kit v3-cBot-HS (Illumina) and sequenced on an Illumina Hiseq 2000 platform. Finally, 100 bp paired-end reads were generated. After removing the reads with adapters, any reads containing ‘n’ (>10%), low quality reads (sQ≤5) and the redundant reads, the remaining clean reads were assembled by TRINITY method [14], and then the redundant contigs were screened by CAP3 [15]. Finally, the unigenes were searched against Nr database (NCBI non-redundant protein sequences) by Blast2GO [16], and the orthologs were used as the reference sequences. All cDNA data series were submitted to NCBI Sequence Read Archive (SRA) database with accession number SRR1131121.

### Construction and high-throughput sequencing of Small RNA library

According to the protocol of Illumina TruSeq Small RNA Sample Preparation Kit (Illumina), 3 ug pooled RNA was used as small RNA library construction. In brief, RNA bands around 20–30 bp were separated and purified by 6% TBE PAGE gel and subsequently bound to 3′ and 5′ end adapters in two separated subsequent steps, which followed by PAGE gel purification. After the first strand cDNA synthesizing by random oligonucleotides and SuperScript II and amplifying by PCR, DNA fragments ligated with adapters on both ends were selectively enriched using Illumina PCR Primer Cocktail in a 12 cycles PCR reaction, and the products of 145 bp to 160 bp (with adaptors on both sides) were separated by PAGE gel, and quantified by Agilent 2100 bioanalyzer. Then, the cluster of index-coded samples was generated using TruSeq SE Cluster Kit v3-cBot-HS (Illumina) and sequenced on an Illumina Hiseq 2000 platform. Finally, 50 bp single-end reads were generated. All small RNA data series were submitted to SRA database with accession number SRR1129970.

### Filter of small RNA reads and microRNAs identification

After removing the unclean reads (the adapters, low quality reads, reads containing ‘n’, and redundant reads), clean unique reads were mapped onto the *A. schrenckii* transcriptome reference sequences using the program Bowtie [17] with no mismatch. Perfectly mapped reads were scanned against the Metazoa mature microRNA (miRNA) of Sanger miRBase (Release 19) [18] to identify the orthologs of known miRNAs. Then, the non-conserved unique reads were screened against Rfam (http://rfam.sanger.ac.uk/) [19] and RepeatMasker (http://www.repeatmasker.org/) [20] successively using the program Bowtie to filter the sequences originating from rRNA, tRNA, snRNA, snoRNA and repetitive elements.

The potential miRNA reads, which were unannotated small RNA tags and could be mapped onto the transcriptome reference sequences, were analyzed by miREvo [21] and mirdeep2 [22] for the prediction of Dicer cleavage site, the assay of secondary structure and the minimum free energy. Finally, the potential miRNA candidates were submitted to miRBase again, and the precursors (hairpins) of potential miRNAs that passed MirCheck [23] were manually inspected the canonical structure of miRNAs in order to remove the false prediction. The base bias of mature miRNAs on the first nucleotide position with certain length and on each position of all identified miRNAs (>100 reads) were calculated, respectively.

### MiRNA microarray and data analysis

We used another 5-month-old Amur sturgeon individual from the Engineering and Technology Center of Sturgeon Breeding and Cultivation of Chinese Academy of Fishery Science (Beijing, China) to validate the expression of 103 miRNAs identified by Illumina TruSeq sequencing. The five tissues (liver, spleen, muscle, heart, brain) were sampled and total RNA were extracted as described above. 4 ug total RNA of each sample was used to hybridize with microarray chip.

MiRNA microarray was manufactured by LC Sciences (China), and each miRNA probe has five replicates. The chip was hybridized with RNAs of five Amur sturgeon tissues, which were 3′-extended with a poly (A) tail and ligated with an oligonucleotide tag. Hybridization was performed overnight on a µParaflo microfluidic chip using a micro-circulation pump (Atactic Technologies) [24]. Single-color labeling (Cy3) and hybridization of total RNA were performed according to the manufacture’s protocol with no modification. Microarray results were extracted using a laser scanner (GenePix 4000B, Molecular Device) and digitized using Array-Pro image analysis software (Media Cybernetics). Raw data were subtracted by the background matrix, and then normalized using a LOWESS (Locally-weighted Regression) method to remove system related variations, including sample amount variations and signal gain differences of scanners, and thus faithfully reveal the biological variations [25]. The transcripts will be defined as detectable when their signal intensity higher than 3× (background standard deviation), spot CV [(standard deviation)/(signal intensity)]<0.5, and 50% of the repeating probes are meet the first two criteria. Finally, the expression of miRNAs was determined with the criterion of signal intensity and *P* value. The microarray data were deposited in a database (ArrayExpress, GEO) with accession number GSE57102.

### Quantitative miRNA real-time PCR assay

Stem-loop reverse transcription (RT) real-time PCR was used to quantify the expression of ten mature miRNAs in five tissues (brain, heart, liver, spleen and muscle) of three 5-month-old Amur sturgeon individuals. U6 snRNA, which has relatively stable expression in most of the tissues, was used as the endogenous control [26]. Briefly, 500 ng of total RNA of each sample was reverse-transcribed with miRNA-specific stem-loop RT primers using the First-strand cDNA Synthesis Kit (Thermo Scientific Fermentas). The reactions were incubated at 42°C for 60 min, at 70°C for 15 min and then held at 4°C. Real-time PCR was performed in triplicate wells using Strategene Mx3000P (Agilent Technologies company, American) according to the protocol. In a 20 µl reaction mixture, 2.0 µl of cDNA was used as template, with 10 µl of SYBR Select Master Mix (Applied Biosystems, Carlsbad, USA), 1.0 µl of specific forward primer, and 1.0 µl of universal primer, with the following program: 50°C for 2 min for UDG (Heated-labile Uracil-DNA Glycocasylase) activation and then 95°C for 2 min, followed by 40 cycles of 95°C for 15 s, 60°C for 30 s and 72°C for 30 s. Primers used in this study were listed in S1 Table. The 2^−ΔCT^ method was used to calculate the relative expression (versus U6 snRNA).

### MiRNA targets prediction and annotation

Hereafter, we only considered the miRNAs detected by microarray with the criteria of Signal >500 and *P*≤0.01 for further analysis.

For miRNAs target gene prediction, we first predicted the Open Reading Frame (ORF) of Amur sturgeon reference sequences, identified the orthologous mRNAs and then predicted the 3′UTR by searching against the vertebrate genomic database in GENSCAN (http://genes.mit.edu/GENSCAN.html). The 3′ UTR sequences of the orthologs were trimmed and analyzed by using the Perl scripts of both TargetScan v6.2 with context score percentile ≥50 (http://www.targetscan.org/) and miRanda v3.3a with Max_Energy ≤ −20 (http://www.microrna.org/microrna/home.do) for the target gene prediction. Then, the target genes were annotated by mapping to Gene Ontology (GO) database (http://www.geneontology.org/) and KEGG pathways database (http://www.genome.jp/kegg/) by BLASTX at E values ≤1e-5 [27]. Finally, the enriched functional groups or pathways among miRNA putative targets were identified with *P*<0.05.

## Results

### Transcriptome references sequencing

A total of 5.89×10^7^ reads were sequenced from the ASY transcriptome library with error rate of 0.04%, Q30 of 87.52% and GC content of 49.61%. Total 5.01×10^7^ (85.07%) clean reads remained after removing the low quality and contaminant reads (S1 Figure). After reads assembly, removing the redundancy and annotation of unique sequences, a total of 148,817 unigenes (N_50_ = 1599) specific to *Acipenser schrenckii* were obtained, including 41,378 protein-coding sequences.

### Sequencing and statistics of small RNA reads

A total of 1.67×10^7^ reads were sequenced from the ASY small RNA library with error rate of 0.01%, Q30 of 94.14% and GC content of 53.61%. Then a total 1.35×10^7^ high-quality small RNA reads were obtained after removing the ambiguous reads (Table 1). The size distribution and frequency percentage of small RNA reads are shown in S2 Figure, and in them, the potential miRNA reads (21–24 bp) were the major part (about 44%).

**Table 1:** Classification of small RNA reads of *Acipenser schrenckii*.

Classification	Number of reads	Percentage (%)
Total Small RNA Reads	16728944	100
Total Clean Small RNA Reads	16197616	96.82
Total Unique Small RNA Reads	13538171	80.93
Total Perfect Matched small RNA reads	8370997	50.04 (100)
Conserved-miRNAs^a^	550453	6.58
rRNA^b^	1771339	21.16
tRNA^b^	24218	0.29
snoRNA^b^	4948	0.06
snRNA^b^	18606	0.22
Repeat^c^	13077	0.16
Novel_miRNA^d^	63555	0.76
Others	5924801	70.78

a miRNA reads identified by searching against the Sanger miRBase (Release 19).

b Screening against the Rfam database to identify the noncoding RNAs using the program Bowtie (-v 0 -k 1).

c Screening against the RepeatMasker database to identify the repeat sequences using the program Bowtie (-v 0 -k 1).

d Novel miRNAs were identified by miREvo and mirdeep2 (-i -r -M -m -k -p 10 -g 50000; quantifier.pl -p -m -r -y -g 0 -T 10).

After mapping to the *A. schrenckii* transcriptome reference sequences, 8.37×10^6^ perfectly matched small RNA reads remained. A total of 5.5×10^5^ reads, were identified by searching against miRBase, corresponding to 6.58% perfectly matched small RNA reads (Table 1). Subsequent small RNAs filter showed that other non-coding RNAs (rRNA, tRNA, snRNA and snoRNA), repeat sequences and unknown genomic regions were about 21.73%, 0.16% and 70.78%, respectively. In those, the repeat sequences were further inspected, and DNA (DNA transposons), minisatellite, LTR (Long Terminal Repeats) and LINE (Long INterspersed Elements) are the most abundant parts (S3 Figure). Finally, 6.4×10^4^ (0.76%) potential novel miRNA reads specific to *A. schrenckii* were detected by miREvo (Table 1).

### MiRNA identification

**Figure 1:**
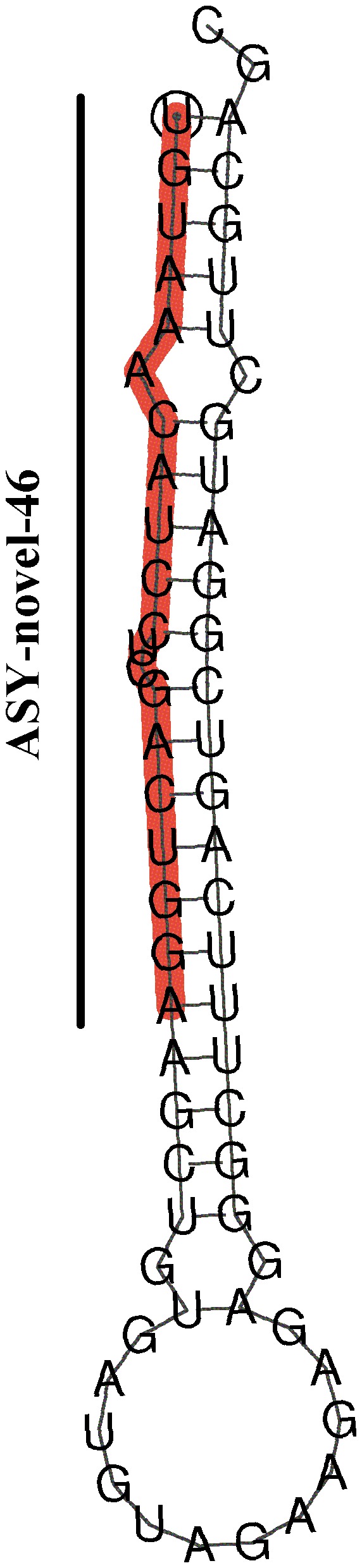
Secondary structure of the novel miRNA (ASY-novel-46) specific to *Acipenser schrenckii* as predicted by miRDeep2.

After the secondary structures analysis and manual examination, we identified 75 miRNA precursors with the stem-loop hairpin structures characteristic to miRNAs precursors, corresponding to 52 unique mature miRNAs named with ASY-X where Xs are numeric numbers (Table 2 and S2 Table). To predict putative miRNAs, we applied miRDeep2 program, which incorporates the position and frequency of small RNAs with the secondary structure of miRNA precursor and can discover novel miRNAs [28], [29], on the potential novel miRNA reads specific to *A. schrenckii*. We obtained 51 unique miRNA precursors, corresponding to 51 uniquely putative novel miRNAs named ASY-novel-X (Table 2 and S2 Table). In those, three putative novel miRNAs were found in miRBase (ASY-novel-5 and ASY-novel-51 are identical with pol-miR-144-5p and pol-miR-133-5p respectively; ASY-novel-37 is similar to dre-miR-1306 with one mismatch). Thus, these three miRNAs were classified into the conserved miRNAs and named as ASY-miR-144-5p, ASY-miR-133-5p and ASY-miR-1306. The secondary structure of most abundant putative novel miRNA (ASY-novel-46) is shown in Fig. 1.

**Table 2:** Top 20 highest expressed miRNAs of *Acipenser schrenckii* identified by Illumina sequencing.

ASY_id	Sequence	Read counts
ASY-miR-21	UAGCUUAUCAGACUGGUGUUGGC	251614
ASY-miR-133a-3p-1	UUUGGUCCCCUUCAACCAGCUG	55575
ASY-miR-133a-3p	UUGGUCCCCUUCAACCAGCUGU	55571
ASY-miR-9-5p-1	UCUUUGGUUAUCUAGCUGUAUGA	53714
ASY-miR-9-3p	UCUUUGGUUAUCUAGCUGUAUG	53714
ASY-let-7a	UGAGGUAGUAGGUUGUAUAGUU	51494
ASY-novel-46	UGUAAACAUCCUCGACUGGA	39788
ASY-miR-181b-1	AACAUUCAUUGCUGUCGGUGGG	23528
ASY-miR-181b	AACAUUCAUUGCUGUCGGUGG	23512
ASY-let-7e	UGAGGUAGUAGAUUGAAUAGUU	18018
ASY-miR-142a-5p	CAUAAAGUAGAAAGCACUACU	13242
ASY-miR-126a-3p	UCGUACCGUGAGUAAUAAUGC	10735
ASY-miR-126-3p	CUCGUACCGUGAGUAAUAAUGC	10735
ASY-miR-499	UUAAGACUUGCAGUGAUGUUUA	10246
ASY-miR-101a	UACAGUACUGUGAUAACUGAAG	7693
ASY-miR-128-1	UCACAGUGAACCGGUCUCUUUU	7487
ASY-miR-128	UCACAGUGAACCGGUCUCUUU	7487
ASY-miR-199-3p-1	UACAGUAGUCUGCACAUUGGUU	6583
ASY-miR-199-3p	ACAGUAGUCUGCACAUUGGUU	6576
ASY-novel-1	CAUUAUUACUGUUGGUACGCG	5950

ASY_id, *A. schrenckii* mature miRNAs ID annotated as described in the text. For detailed information about sturgeon miRNAs please see S2 Table.

**Figure 2:**
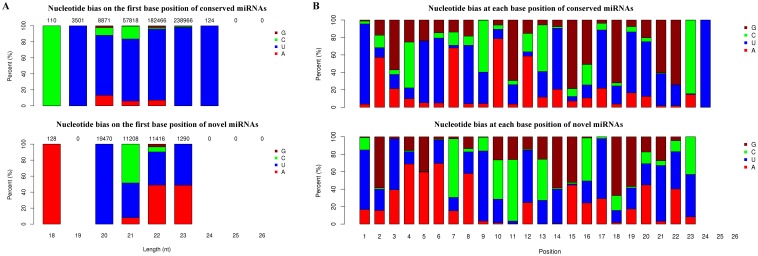
Base bias analysis of the first nucleotide of *Acipenser schrenckii* miRNAs. (A) Base bias percentage on the first position of conserved miRNAs (top) and novel miRNAs specific to *A. schrenckii* (bottom) sized from 18 ∼26 bp. The x-axis indicates the length of miRNAs. The y-axis indicates the percentage of four nucleotide acids. The numbers on the columns are the total numbers of miRNAs with specific length of each nucleotide. (B) Base bias percentage from the first to the 26th base pairs of conserved miRNAs (top) and novel miRNAs (bottom). The x-axis indicates the location of base pairs. The y-axis indicates the percentage of four nucleotide acids.

The base-bias analysis indicated the strong preference of nucleotide utility in both conserved and putative novel miRNAs (Fig. 2A and 2B). Among conserved miRNAs, the preference of first base utility shows a close correlation with length of miRNAs. The miRNAs with the length of 18 bp prefer the nucleotide C at the first base position, those of 19–24 bp favor the U (Fig. 2A, top). Similarly, the first base utility of putative novel miRNAs prefer the A in those with 18 bp length and the U in those with the length of 20 bp, whereas others with length of 21–23 bp favor the use of C/U or A/U (Fig. 2A, bottom). Moreover, we found that the nucleotide utility shows difference on each site position between conserved and putative novel miRNAs. For conserved miRNAs, the nucleotide U has the most frequency among sites, then followed by G, A and C (Fig. 2B, top). Whereas, the nucleotide A has the highest utility rate among putative novel miRNAs, and other three nucleotides (G, C and U) share the relative equal utility (Fig. 2B, bottom).

### Expression pattern assay of miRNAs by microarray and real-time PCR

We used the independent microarray platform to validate the expression level of 103 miRNAs obtained by Illumina TruSeq sequencing. A total of 87 miRNAs were detected by microarray in at least one of five tissues (S3 Table), and in those, 57 miRNAs have strong detected signal with the criterion of Signal >500 and *P*<0.01; 14 miRNAs had lower signal with *P*<0.01, and Signal <500 (S3 Table). Moreover, we found ASY-miR-21, the highest expressed miRNA in TruSeq, had detectable signal with Signal = 2.5×10^5^ and *P* = 0.0108; ASY-novel-1 with high expressed level in TruSeq had low detectable signal (Signal <500), and *P*<0.01 (S3 Table).

**Figure 3:**
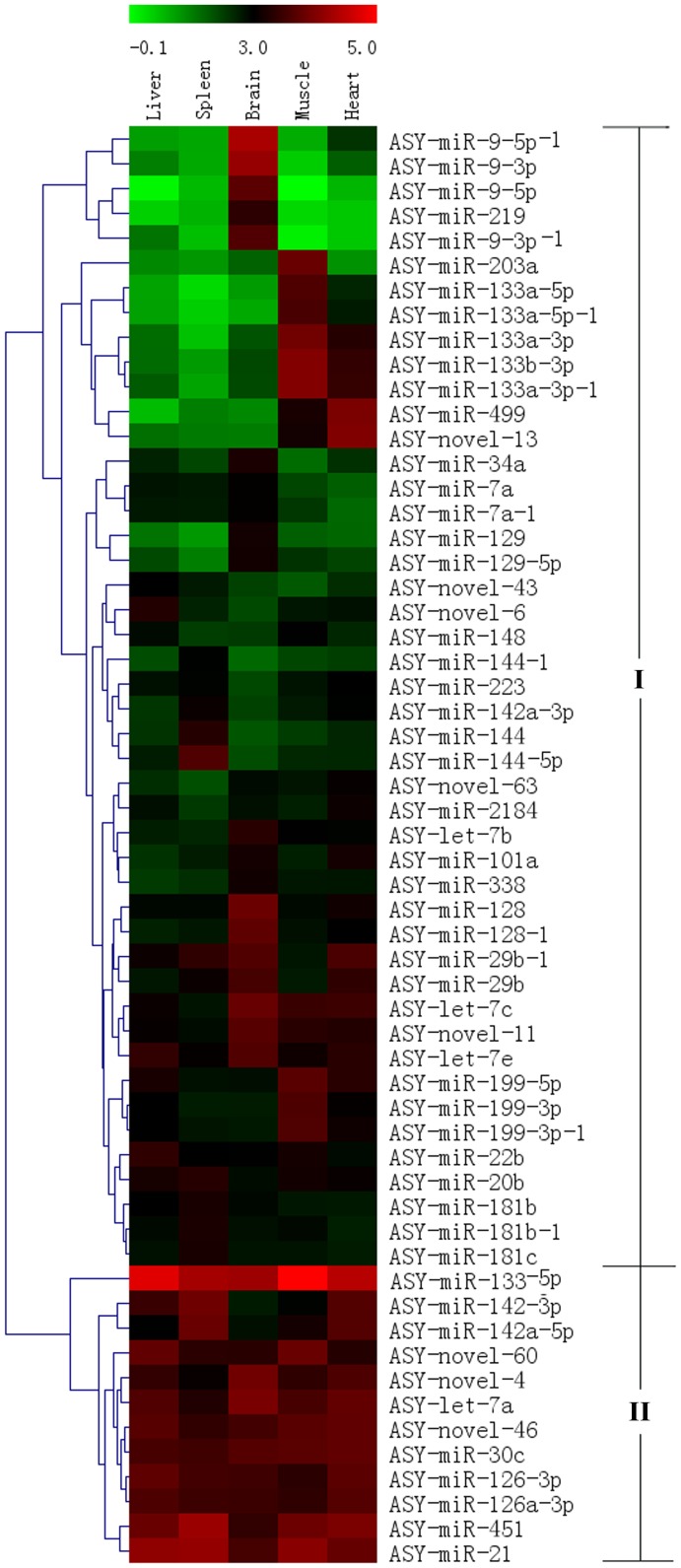
Expressed pattern of miRNAs in five tissues of *Acipenser schrenckii*. The expression of 58 miRNAs detected by microarray (Signal >500 and *P*≤0.01) are reflected by Log-normalized intensities. Heat map represents the miRNAs which were clustered into two clades based on their expression in tissues.

Further analysis indicated that, in 58 miRNAs with high detected signal (Signal >500 and *P*≤0.01), 21 miRNAs showed co-expression in all five tissues, and 16 miRNAs (one in liver, three in spleen, three in muscle and nine in brain) highly expressed in one specific tissue (Table 3). The expressed pattern of 58 miRNAs (Signal >500 and *P*≤0.01) in five tissues indicated that miRNAs were mainly separated into two clades, clade I, 46 of these are expressed at low levels in most of tissues, however, some of these are highly expressed in specific tissues such as brain, muscle and heart; and clade II, 12 miRNAs are very highly expressed in most of five tissues (Fig. 3).

**Table 3:** Details of miRNAs co-expressed in five tissues and specifically high-expressed in one tissue obtained by microarray.

ASY id	Liver	Spleen	Brain	Muscle	Heart	*P* value
**Co-expressed in five tissues**						
ASY-novel-51	**58866**	**20014**	**17266**	**178365**	**28239**	**0**
ASY-miR-451	**6815.4**	**16822**	**2485.9**	**7241.7**	**9525.7**	**0**
ASY-miR-21	**13638**	**14762**	**3603.1**	**11996**	**6257.9**	**0.010779**
ASY-let-7a	**4341.2**	**1829**	**9101.5**	**3628.9**	**6001.2**	**1.19E-06**
ASY-miR-30c	**3620.8**	**3191.8**	**4799.2**	**5151.8**	**5884.4**	**0.004648**
ASY-novel-46	**4824**	**2462.3**	**3405.9**	**4993.2**	**5823.8**	**1.16E-13**
ASY-miR-126-3p	**5670.9**	**3472.9**	**3121.6**	**2181.5**	**5295.7**	**8.14E-12**
ASY-miR-126a-3p	**4029.2**	**3090.4**	**2907.8**	**2341.1**	**4654.2**	**0.006502**
ASY-miR-142a-5p	**950.58**	**6718.8**	**647.5**	**1484.2**	**4488.5**	**2.03E-12**
ASY-novel-4	**2529.3**	**1178.8**	**7718.6**	**2370.6**	**4046.6**	**1.09E-12**
ASY-miR-29b-1	**1279.1**	**2379.8**	**4467.7**	**545.06**	**3926.8**	**1.20E-12**
ASY-let-7c	**1254.1**	**579.54**	**6588.6**	**2874.6**	**3028.3**	**0**
ASY-let-7e	**2467.7**	**1098.1**	**4410.8**	**1345**	**2107.8**	**7.87E-06**
ASY-miR-199-5p	**1593.7**	**633.36**	**680.25**	**4990.1**	**2099.6**	**0**
ASY-novel-60	**5910.5**	**2212.4**	**2117.2**	**6668.1**	**2013.5**	**2.86E-12**
ASY-novel-11	**1170.7**	**734.91**	**4854.9**	**2150.9**	**1882.3**	**3.31E-07**
ASY-miR-128	**829.98**	**809.69**	**7035.4**	**764.35**	**1389.7**	**0**
ASY-miR-199-3p-1	**996.7**	**555.67**	**514.27**	**4289.6**	**1333.7**	**1.11E-16**
ASY-miR-20b	**1563.6**	**2048.8**	**717.95**	**1419.8**	**1181.8**	**0.000648**
ASY-miR-22b	**2393.7**	**995.11**	**951.48**	**1493.4**	**750.99**	**8.44E-11**
ASY-miR-181b	**1054.8**	**1592.9**	**822.1**	**529.34**	**511.25**	**5.95E-13**
**Specifically high-expressed in one tissue**						
ASY-novel-43	**1047.8**	486.71	160.39	81.223	289.85	**0**
ASY-novel-5	444.27	**4266.1**	117.68	334.44	339.88	**0.000511**
ASY-miR-144	235.18	**2002.1**	88.552	187.07	345.78	**0**
ASY-miR-144-1	118.14	**944.84**	53.171	139.42	174.44	**5.84E-14**
ASY-miR-9-5p	11.958	9.1725	**21752**	7.7512	237.41	**1.05E-07**
ASY-miR-9-3p	27.953	8.7851	**15396**	3.204	71.675	**6.87E-06**
ASY-miR-9-5p	0.99	5.3945	**5358.4**	0.816	6.1162	**2.88E-06**
ASY-miR-9-3p	39.146	4.8833	**4368**	1.187	3.6751	**0.000264**
ASY-miR-219	2.8917	6.5155	**2291.8**	2.3486	3.6736	**6.63E-05**
ASY-miR-34a	378.58	138.9	**1655.4**	47.675	267.51	**1.11E-16**
ASY-miR-129	39.144	14.056	**1442.2**	68.223	56.267	**2.73E-11**
ASY-miR-129-5p	127.43	28.697	**1412.8**	241.71	147.94	**5.25E-07**
ASY-miR-7a-1	499.3	479.58	**1086.1**	217.23	53.966	**3.68E-12**
ASY-miR-203a	21.864	14.346	62.816	**6787.7**	16.729	**2.91E-06**
ASY-miR-133a-5p	10.227	2.2293	12.82	**4165.2**	349.34	**6.05E-09**
ASY-miR-133a-5p-1	10.976	3.1048	8.7457	**3750**	476.91	**1.32E-06**

Detected signal were shown as means. Signal > 500 was shown in bold.

**Figure 4:**
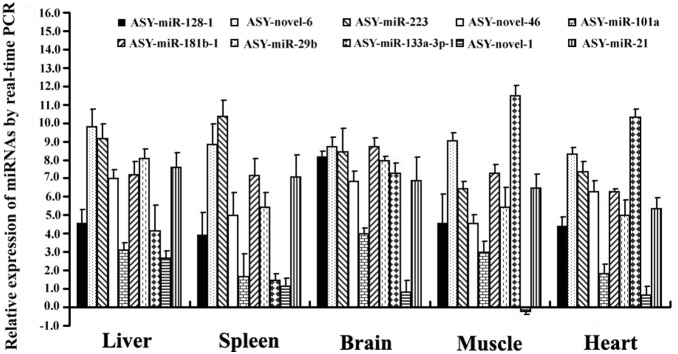
Profiling of ten sturgeon miRNAs expression by real-time PCR. Figure shows the relative expression of miRNAs (vs. U6 snRNA) and transformed by Log2. Data are presented as mean±SD (standard deviation) and n = 3.

We randomly tested the expression of ten miRNAs (seven conserved miRNAs and three novels specific to *A. schrenckii*) in five tissues of three sturgeon individuals respectively by stem-loop real-time PCR assay. All these miRNAs have high expressed levels detected by both Illumina TrueSeq sequencing and microarray, with the exception of ASY-novel-1 which shows low expression level in microarray. Real-time PCR results supported the expression pattern of miRNAs obtained by microarray, with the exception of ASY-miR-101a, which showed much lower expression level in heart than that seen in the microarray (Fig. 3 and Fig. 4).

### Target prediction and annotation

To better understand the functions of miRNAs, the putative targets of 87 miRNAs, which detected by both Illumina TruSeq sequencing and microarray were predicted by TargetScan and miRanda based on the *A. schrenckii* transcriptome. Firstly, in 148,817 of *A. schrenckii* unigenes, a total of 68435 genes, orthologous to vertebrate mRNAs and with the best hit, were remained. In these, 14265 of 68435 *A. schrenckii* unigenes were identified as the targets of miRNAs by both TargetScan (context score percentile≥50) and miRanda (Max_Energy ≤ −20). A total of 3391 GO terms and 228 KEGG pathways regulated by 87 miRNAs were also identified. In those, 369 enriched functional groups and 37 enriched functional KEGG pathways, which were mainly regulated by 58 high-expressed miRNAs, were obtained with *P*<0.05 (S4 Table). Most of the enriched functional pathways or groups were involved in cell development, signal transduction, metabolic and immune processes.

## Discussion

Our study shows that total 58 of 103 microRNAs (miRNAs) were confirmed by both Illumina TruSeq sequencing and microarray in Amur sturgeon *Acipenser schrenckii*, including 21 miRNAs co-expressed in all five tissues and 16 which have tissue-specific expression. According to the functional annotation and the enrichment analysis of putative targets, these miRNAs are mainly involved in development, metabolism, immune response and gametogenesis.

For small RNA filtration and miRNA annotation, we firstly performed the transcriptome reference sequencing by Illumian TruSeq platform. Total 148,817 unigenes, which are assembled by 5.01×10^7^ clean reads and specific to *A. schrenckii*, were obtained. By small RNA sequencing, we obtained a total of 1.35×10^7^ high-quality small RNA reads and of them, about 44% are the potential miRNA reads with 21–24 bp in length (Table 1 and S2 Figure). After mapping to the *A. schrenckii* transcriptome reference sequences, about 7% reads were identified as potential miRNAs by searching against miRBase and miREvo detection, and the unknown genomic regions were the major part about 70%. The small mapping proportion of potential miRNA reads and a large number of unknown genomic sequences in small RNAs reads of sturgeon are not unique, whereas have been observed in some organisms, such as ∼3% vs. ∼94% in sea cucumber [12] and ∼1% vs. ∼97% in swithgrass [30]. This result may because of the lack of genomic background and limited transcriptome information specific to the Amur sturgeon. Moreover, another reason there is a low amount of small RNA reads is the degradation of RNA sample, which is indicated by the high proportion of rRNA (∼21%). In this study, we conducted the TruSeq sequencing with RNA pool of five tissues (RIN = 8.3), whereas, we did not test the RIN of five RNA samples separately before pooling samples. Thus, there is a chance that some sample degradation may have happened before pooling.

Our data also revealed a large number of sturgeon specific miRNAs, and provided candidates for further study of sturgeon biology. A total of 103 mature miRNAs were identified by TruSeq, including 55 conserved miRNAs and 48 novel miRNAs (S2 Table). Further analysis suggested the strong preference of nucleotide utility of sturgeon miRNAs, and also the great difference of nucleotide utility between conserved and novel miRNAs, especially in the first base position (Fig. 2). Previous studies have shown that the first nucleotide at the 5′ end of miRNA is considered key for strand selectivity of Dicer-mediated cleavage [31]. The strong preference of nucleotide utility on the first base position of sturgeon miRNAs may affect the Dicer cleavage, and thus, the target recognition [31], [32]. Further comparing the preference of nucleotide utility of sturgeon miRNAs with those of other organisms will increase the understanding on the interaction of sturgeon miRNAs-mRNAs.

Of 103 sturgeon miRNAs, 87 were detected by microarray validation including 58 miRNAs with strongly detected signals (S3 Table). Moreover, of those miRNAs with strongly detected signals, we randomly tested the expression pattern of 10 miRNAs in five sturgeon tissues by real-time PCR, and obtained consistent results with that of microarray (Fig. 3 and Fig. 4). In addition, we found that 21 miRNAs were co-expressed in all five tissues, and this suggested the crucial role of them for sturgeon physiological processes (Table 3). In them, 8 miRNAs (miR-21, miR-30c, miR-126-3p, let-7c, let-7e, miR-128, miR-20b and miR-181b) had been shown to be related with gametogenesis [8], [33]. Furthermore, 16 miRNAs were tissue-specifically expressed (Table 3), indicated the particular roles of these miRNAs in the related tissues. MiR-144, with the co-regulation of miR-451, has been proven to be involved in many diseases, such as anemia severity of sickle cell disease [34], cancer [35], brain aging and spinocerebellular ataxia pathogenesis [36]. The high-expression of miR-144 specifically in spleen, an immune and hematopoietic organ, and also miR-451 in all five tissues of sturgeon suggest the key role of miR-144/451 in sturgeon immune system. The specific high-expression of miR-133a in muscle, which has function in the proliferation and differentiation of cardiomyocytes, bronchial smooth muscle and related diseases [37]–[39], implies the role of miR-133a in sturgeon physiological processes. Moreover, studies showed that miR-9 plays role in neural diseases and cancers [40]–[42], miR-219 regulates neural precursor maintenance and specification [43], miR-34a is a tumor suppressor [44], miR-129 promotes apoptosis [45], and miR-7a regulates the neuronal excitability [46], and all these miRNAs were found to be specifically expressed in sturgeon brain. The identification of tissue-specifically expressed miRNAs, combining with the co-expressed miRNAs in all five tissues, provides clues to further study the molecular mechanism of sturgeon physiological processes.

KEGG pathway and GO annotation analyses could provide a better understanding of the potential functions of miRNAs by illustrating the function of target mRNAs. Because of the absence of the genomic information and incomplete transcriptome annotation of sturgeon, we used the genomic sequences of all vertebrate species deposited in the online gene analysis software (GENSCAN) as reference to predict the target gene of Amur sturgeon miRNAs. The GO terms and KEGG pathways identified here (S4 Table), especially the enriched functional groups and pathways which regulated by 58 miRNAs with strongly detected signal, provide us with guidance to purposely comb the miRNAs and putative mRNAs from the complex gene database networks, and can be used in the future for sturgeon aquaculture.

With the progress of the sturgeon genomic and transcriptome information, or for other closer species that become available, the accuracy of sturgeon gene annotation will be increased and much more detailed information of *A. schrenckii* will be uncovered from our mRNA and miRNA transcriptome datasets. Furthermore, compared with other teleost aquaculture species, the long lifespan and time to breeding capability of the sturgeon results in high maintenance costs (eg. space, energy consumption and water purification). MiRNAs that may be involved in cell differentiation and development, signal transduction, gametogenesis, metabolic and immune processes, and their targets provide opportunity for early sex determination and also to select individuals with rapid growth and disease resistance for breeding purposes.

## Conclusions

This study reveals the first sturgeon miRNAs profile, and the findings advance the understanding of sturgeon biology and are valuable for sturgeon fishery and conservation. However, the validation of the relationship between sturgeon miRNAs and target mRNAs in the regulation of specific physiological processes needs further biologically experimental evidences.

## Supporting Information

S1 Figure **Overview of** ***Acipenser schrenckii*** **transcriptome sequencing reads.**
(PNG)

S2 Figure **The sequence length distribution and frequence percentage of small RNA reads of** ***Acipenser schrenckii*** **.** The x-axis indicates the length of small RNA reads. The y-axis indicates the percentage of small RNA reads with specific length. Different color suggests different type of small RNAs.
(PNG)

S3 Figure **Classification of repeat sequences of** ***Acipenser schrenckii*** **small RNA library.** Ambi: ambiguous reads; RC, rolling circle; LINE, Long INterspersed Elements; SINE, Short INterspersed Elements; LTR, Transposable elements with Long Terminal Repeats; DNA, DNA transposons. +, sense strand; -, anti-sense strand.
(PNG)

S1 Table **Forward, stem-loop and universal primers used to amplify miRNAs and U6 snRNA in real-time PCR.**
(DOC)

S2 Table **Details of mature miRNAs and hairpins screening by Illumina.** (A) 55 conserved mature miRNAs; (B) 75 conserved miRNA hairpins; (C) 48 mature novel miRNAs specific to *A. schrenckii*; (D) 51 noval miRNA hairpins.
(XLSX)

S3 Table **Summary of 87 miRNAs identified by both Illumina TruSeq sequencing and microarray.** RPM, Reads Per Million.
(XLS)

S4 Table **Details of GO annotation and KEGG pathway analysis.** The enriched functional groups and pathways were identified by Fisher’s Exact Test (*P*<0.05). S gene number: Number of Significant genes matched to single GO term or KEGG pathway; TS gene number: Total number of Significant genes matched to GO terms or KEGG pathways; B gene number: Number of genes matched to single GO term or KEGG pathway; TB gene number: Total number of genes matched to GO terms or KEGG pathways.
(XLS)
