# Supplementary figures and images for: High-Throughput Sequencing of MicroRNA Transcriptome and Expression Assay in the Sturgeon, Acipenser schrenckii
Source: PLoS One. 2014 Dec 15;9(12):e115251. doi: 10.1371/journal.pone.0115251 (PMC4266654; doi:10.1371/journal.pone.0115251)

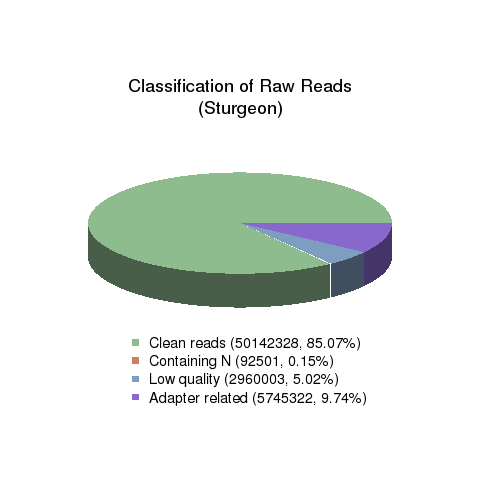

Supplement: S1 Figure — Overview of Acipenser schrenckii transcriptome sequencing reads. (PNG) [file pone.0115251.s001.png]

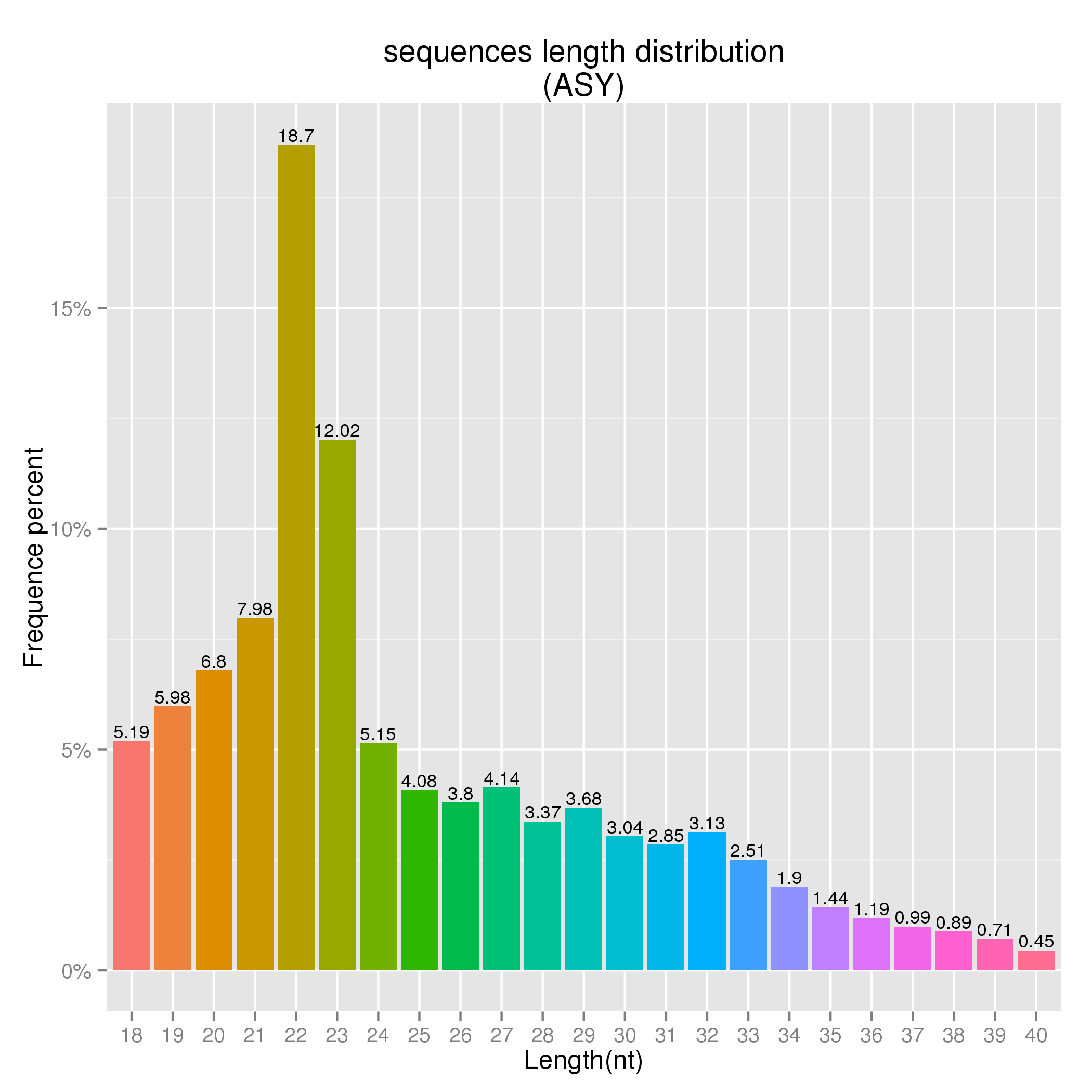

Supplement: S2 Figure — The sequence length distribution and frequence percentage of small RNA reads of Acipenser schrenckii . The x-axis indicates the length of small RNA reads. The y-axis indicates the percentage of small RNA reads with specific length. Different color suggests different type of small RNAs. (PNG) [file pone.0115251.s002.png]

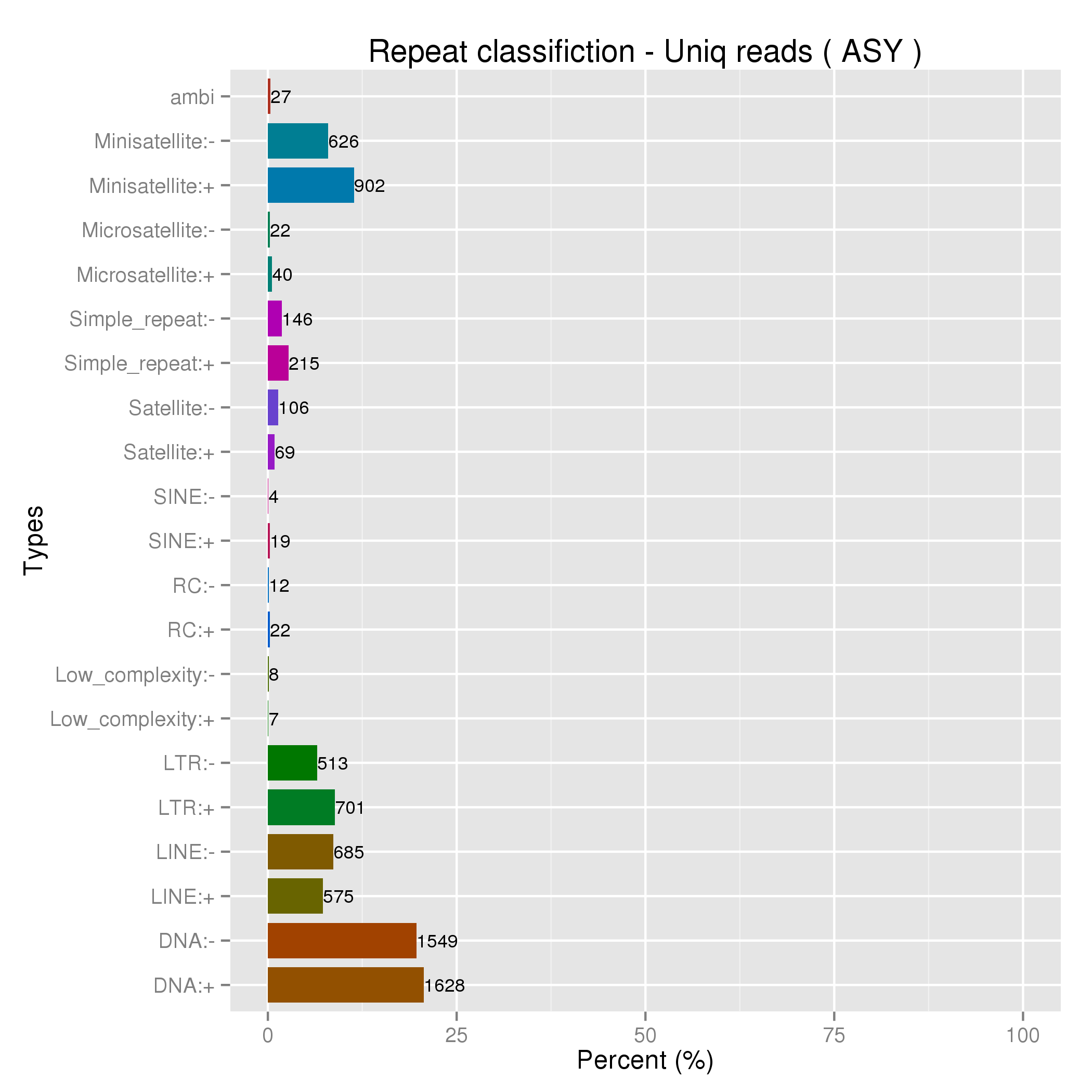

Supplement: S3 Figure — Classification of repeat sequences of Acipenser schrenckii small RNA library. Ambi: ambiguous reads; RC, rolling circle; LINE, Long INterspersed Elements; SINE, Short INterspersed Elements; LTR, Transposable elements with Long Terminal Repeats; DNA, DNA transposons. +, sense strand; -, anti-sense strand. (PNG) [file pone.0115251.s003.png]
