# Supplementary material for: High-Throughput Sequencing of MicroRNA Transcriptome and Expression Assay in the Sturgeon, Acipenser schrenckii
Source: PLoS One. 2014 Dec 15;9(12):e115251. doi: 10.1371/journal.pone.0115251 (PMC4266654; doi:10.1371/journal.pone.0115251)
Supplement: S1 Table — Forward, stem-loop and universal primers used to amplify miRNAs and U6 snRNA in real-time PCR. (DOC) [file pone.0115251.s004.doc]

**Table S1. Forward, stem-loop and universal primers used to amplify miRNAs and U6 snRNA in real-time PCR.**

| **Primer** |  | **Sequence** |
| --- | --- | --- |
| **ASY-miR-21** | **Stem**  **Fwd**  **Rev** | 5’-GTCGTATCCAGTGCAGGGTCCGAGGTATTCGCACTGGATACGACGCCAAC -3’  5’- GCCGTAGCTTATCAGACTGG-3’  5’-GTGCAGGGTCCGAGGT-3’ |
| **ASY-miR-181b-1** | **Stem**  **Fwd**  **Rev** | 5’-CTCACAGTACGTTGGTATCCTTGTGATGTTCGATGCCATATTGTACTGTGAGCCCACCGA -3’  5’-ACACTCCAGCTGGGAACATTCATTGCTGTCGGT-3’  5’-CTCACAGTACGTTGGTATCCTTGTG-3’ |
| **ASY-miR-29b** | **Stem**  **Fwd**  **Rev** | 5’-CTCACAGTACGTTGGTATCCTTGTGATGTTCGATGCCATATTGTACTGTGAGAACACTG -3’  5’ - ACACTCCAGCTGGGTAGCACCATTTGAAATCAG -3’  5’-CTCACAGTACGTTGGTATCCTTGTG-3’ |
| **ASY-miR-128-1** | **Stem**  **Fwd**  **Rev** | 5’-CTCACAGTACGTTGGTATCCTTGTGATGTTCGATGCCATATTGTACTGTGAGAAAAGAG -3’  5’-ACACTCCAGCTGGGTCACAGTGAACCGGTCTCT-3’  5’-CTCACAGTACGTTGGTATCCTTGTG-3’ |
| **ASY-miR-223** | **Stem**  **Fwd**  **Rev** | 5’-CTCACAGTACGTTGGTATCCTTGTGATGTTCGATGCCATATTGTACTGTGAGGGGGTAT -3’  5’-ACACTCCAGCTGGGTGTCAGTTTGTCAAATAC-3’  5’-CTCACAGTACGTTGGTATCCTTGTG-3’ |
| **ASY-miR-133a-3p-1** | **Stem**  **Fwd**  **Rev** | 5’-CTCACAGTACGTTGGTATCCTTGTGATGTTCGATGCCATATTGTACTGTGAGCAGCTGGT -3’  5’-ACACTCCAGCTGGGTTTGGTCCCCTTCAACCAG-3’  5’-CTCACAGTACGTTGGTATCCTTGTG-3’ |
| **ASY-miR-101a** | **Stem**  **Fwd**  **Rev** | 5’-CTCACAGTACGTTGGTATCCTTGTGATGTTCGATGCCATATTGTACTGTGAGCTTCAGT -3’  5’-ACACTCCAGCTGGGTACAGTACTGTGATAACT-3’  5’-CTCACAGTACGTTGGTATCCTTGTG-3’ |
| **ASY-novel-1** | **Stem**  **Fwd**  **Rev** | 5’-CTCACAGTACGTTGGTATCCTTGTGATGTTCGATGCCATATTGTACTGTGAGCGCGTAC -3’  5’-ACACTCCAGCTGGGCATTATTACTGTTGGTAC-3’  5’-CTCACAGTACGTTGGTATCCTTGTG-3’ |
| **ASY-novel-6** | **Stem**  **Fwd**  **Rev** | 5’-CTCACAGTACGTTGGTATCCTTGTGATGTTCGATGCCATATTGTACTGTGAGGCCTGTG -3’  5’-ACACTCCAGCTGGGTGATAGAACTCCGTGCCA-3’  5’-CTCACAGTACGTTGGTATCCTTGTG-3’ |
| **ASY-novel-46** | **Stem**  **Fwd**  **Rev** | 5’-CTCACAGTACGTTGGTATCCTTGTGATGTTCGATGCCATATTGTACTGTGAGTCCAGTC -3’  5’-ACACTCCAGCTGGGTGTAAACATCCTCGACT-3’  5’-CTCACAGTACGTTGGTATCCTTGTG-3’ |
| **U6 snRNA** | **Fwd**  **Rev** | 5’-CGCTTCGGCAGCACATATAC-3’  5’-TTCACGAATTTGCGTGTCA-3’ |
